# Editorial: Zebrafish Cognition and Behavior

**DOI:** 10.3389/fnbeh.2021.659501

**Published:** 2021-03-16

**Authors:** Ana Carolina Luchiari, Edward Málaga-Trillo, Steven Tran, Robert Gerlai

**Affiliations:** ^1^Department of Physiology and Behavior, Federal University of Rio Grande Do Norte, Natal, Brazil; ^2^Department of Biology, Universidad Peruana Cayetano Heredia, Lima, Peru; ^3^Department of Biology and Biological Engineering, California Institute of Technology, Pasadena, CA, United States; ^4^Department of Psychology, University of Toronto Mississauga, Mississauga, ON, Canada

**Keywords:** behavior, learning, memory, fish, perception

Understanding animal cognition has been of interest to scientists for well over a century (e.g., Melrose, 1921). Cognition is broadly defined as the neural and behavioral processes associated with the acquisition, retention, and use of information (Dukas, 2004). Since the discovery of multiple memory systems and of the fundamental role of the hippocampus in relational learning and memory in humans (Penfield and Milner, 1958), one exciting focus of study has been to determine how animals encode, transform, compute and manipulate spatial, temporal, and contextual information from their environment, and how this information is utilized to organize behavioral responses (Cook, 1993). Initial studies used simple visual and acoustic stimuli, such as colored lights and distinct sounds. However, the use of such stimuli hindered the study of animal cognition because it did not allow the subjects to fully engage their full information processing capabilities. To address this issue, researchers started using more complex stimuli, such as objects, photos, and videos. These studies demonstrated a higher level of cognitive processing not previously attributed to animals (Dukas, 2004). As the field of learning and memory advanced, studies started to show remarkable similarities between the cognitive processes of animals and humans. Animals have been found to be even able to learn varied and sophisticated concepts, exhibit mental processes, such as symbol coding and organization, to form spatial, temporal, and numerical abstractions and perceive cause and effect relationships (Wynne, 2001).

While it is widely accepted that mammals and birds have the capacity for complex cognitive processing, the cognitive abilities of certain less well-studied species were questioned and some species were assumed to be able to exhibit only simple stimulus-response reflexes. For example, even fundamental cognitive abilities of fish used to be debated with doubts about whether fish could learn and/or remember. Even in the not-so distant past, some assumed that fish were incapable of complex cognitive processing due to their relatively small and simple brain, and similarly, fish were assumed not to experience suffering but only to exhibit reflexive responses to risk and pain (Rose, 2002; Arlinghaus et al., 2007; Rose et al., 2014).

Although the brains of fish are indeed smaller and simpler, the genetic, neuronal, and physiological mechanisms that drive behavioral responses to a variety of stimuli are similar to those observed in mammals (Ito and Yamamoto, 2009; Klee et al., 2012; Gerlai, 2020). Furthermore, homologous brain regions that perform similar functions, for example, in the regulation of emotional states (dorsomedial pallium equivalent to the mammalian pallial amygdala), and learning and memory (dorsolateral pallium equivalent to the mammalian hippocampus) (Vargas et al., 2009) have been identified. Even the assertion that fish lack a brain area homologous to the mammalian cortex appears debatable (Mueller, 2012).

Many studies suggest that fish exhibit complex behavioral responses that cannot be explained as a simple “stimulus-response” reflex (Trotha et al., 2014; Rey et al., 2015). For instance, fish are capable of exhibiting tool-use like behavior (Kuba et al., 2010; Paśko, 2010; Millot et al., 2014; Brown, 2015), spatial learning (Salas et al., 1996; Sison and Gerlai, 2010; Karnik and Gerlai, 2012; Luchiari et al., 2015), counting (Agrillo et al., 2007, 2008, 2009, 2012), and possess long-term memory (Hinz et al., 2013). In fact, some even suggests that given the neurobiological prerequisites, physiological, anatomical, synaptic, and molecular mechanism related characteristics, as well as behavioral level phenomena, fish may have some level of sentience (Gerlai, 2017; Woodruff, 2017; also see Cerqueira et al., 2020).

Among the many fish species that have contributed to the current knowledge on fish cognition and behavior, the zebrafish, a small teleost fish native to South Asia, stands out as an important vertebrate model in biomedical research. Zebrafish have been used in the fields of embryology (Kimmel et al., 1995; Pinheiro-da-Silva and Luchiari, in press), toxicology (Coe et al., 2009; Oliveira et al., 2009; Dai et al., 2014), genetics (Driever et al., 1994; Liu et al., 2019), pharmacology (Goldsmith, 2004; Barros et al., 2008) mainly because this species is believed to offer translational relevance in biomedical research (Kalueff et al., 2014; Stewart et al., 2014). One of the first studies on zebrafish genetics was performed by George Streisinger in the 70's. Streisinger and colleagues from the University of Oregon successfully generated the first homozygous diploid zebrafish clones (Streisinger et al., 1981), one of the earliest registries of a vertebrate clone.

The many advantages of zebrafish drew the attention of other researchers, especially those who were interested in embryonic development. The main advantage for embryologists was that zebrafish embryos are transparent, which allowed visualization of changes in anatomical structures during ontogenesis. An additional attractive aspect of zebrafish development is that it is very fast: the embryos hatch after 3 days post-fertilization. This fast development has been leveraged in a variety of disciplines from genetics to toxicology and teratology.

At present, although precise figures are lacking, an estimated 8 million zebrafish are being used every year in more than 600 laboratories worldwide, making this species one of the most popular laboratory animals for translational research. In 2000, the zebrafish genome was partially sequenced (Barbazuk et al., 2000), allowing more detailed understanding of the genetic similarities between zebrafish and other vertebrates, including humans. With increasing interest from laboratories worldwide on studying zebrafish cognition, the use of zebrafish translational research also increased. Many questions have arisen including how one can improve cognition, whether it be through exercise or pharmacotherapy, and why cognitive function decays under certain situations, such as sleep deprivation or some brain diseases. Although fish are more distantly related to humans compared to other mammalian models, their simple brain possessing numerous evolutionarily conserved features as well as their rich behavioral repertoire make them a powerful animal model for investigating mechanisms underlying complex behaviors, such as learning and memory (Gerlai, 2020).

Several behavioral responses are the result of cognitive processes, which depend upon structural, physiological, and biochemical characteristics of the central nervous system. These characteristics can be investigated at multiple levels of analysis starting with biochemical interactions all the way to the connectome, remodeling of neural pathways. In this regard, the zebrafish offers the complexity of a vertebrate brain combined with the simplicity and practicality of invertebrates which can utilize classical behavioral and electrophysiological and neurobiological studies along with the latest advancement in proteomics and genetics.

The Research Topic “Zebrafish Cognition and Behavior” in Frontiers in Behavioral Neuroscience samples this rich and fast evolving field, and provides examples on how cognitive function may be studied using this simple vertebrate at multiple organizational levels, from molecules to behavior, and from health to pathology. For example, Pita and Fernández-Juricic explores how shoaling, a complex and dynamic behavior, is influenced by a variety of environmental factors, while Facciol and Gerlai reviews the growing literature on the neurobiological mechanisms underlying shoaling and how this behavior may be affected by embryonic alcohol exposure. Menezes et al. study the behavioral consequences of stress induced by exposure of juvenile zebrafish to abnormally aggressive large male zebrafish. Luz et al. shows that pharmacological intervention using a CB1 receptor agonist counteracts acute restraint-stress induced anxiety-like behaviors, oxidative stress and GABA neurotransmitter level decrease in zebrafish. Gusso et al. shows how an environmental toxicant, pyriproxyfen, impairs memory and cortisol levels in zebrafish. Gómez-Laplaza and Gerlai, studies quantity estimation abilities and the role of different features of food items in decision making in fish using the freshwater angelfish. Daniel and Bhat studies correlations between personality and cognitive traits in zebrafish. Last, Buatois and Gerlai explores often controversial questions concerning elemental vs. configural learning and memory in fish, with a particular focus on zebrafish.

## Author Contributions

All authors listed have made a substantial, direct and intellectual contribution to the work, and approved it for publication.

## Conflict of Interest

The authors declare that the research was conducted in the absence of any commercial or financial relationships that could be construed as a potential conflict of interest.
